# Time-dependent bond strength development and pH dynamics of conventional and self-adhesive resin cements for lithium disilicate restorations

**DOI:** 10.3389/froh.2026.1787202

**Published:** 2026-04-30

**Authors:** Mijoo Kim, Jimin Lee, Reuben Kim, Marc Hayashi

**Affiliations:** 1 UCLA Biomaterials and Device Testing Laboratory, UCLA School of Dentistry, Los Angeles, CA, United States; 2 Section of Restorative Dentistry, UCLA School of Dentistry, Los Angeles, CA, United States

**Keywords:** dental cementation, dual-cure polymerization, lithium disilicate, pH changes, resin cements, self-adhesive cement, setting time, shear bond strength

## Abstract

**Introduction:**

This study investigated shear bond strengths of conventional and self-adhesive resin cements bonded to lithium disilicate at different curing and setting times, and examined pH changes during setting to assess potential pulpal effects. We hypothesized that (1) extended light-curing time would improve bond strength, and (2) adequate light curing would accelerate pH neutralization in self-adhesive cements compared to no light curing, and that (3) conventional cements would achieve higher initial bond strengths compared to self-adhesive systems.

**Methods:**

Lithium disilicate disks were etched and cemented with Variolink Esthetic DC (VA), DuoLink Universal (DU), Panavia SA Universal (PA), and RelyX UniCem2 (RE) using 20 s or 60 s light curing. Bond strengths were measured at 5 min, 2 h, and 24 h. pH was monitored during initial 4 min curing and up to 60 h in Hank's Balanced Salt Solution. Mixed-effects models with Sidak's test analyzed data (*P* = 0.05). Ten specimens per group were evaluated (*n* = 10/group).

**Results:**

Bond strength increased significantly over time for all materials (*P* < 0.0001). During early setting (<5 min), DU and RE showed significantly higher bond strengths regardless of curing time, while VA and PA demonstrated lower initial strengths. Extended curing (60 s) improved final bond strengths. Conventional cements (VA, DU) reached maximum strength within 2 h vs. self-adhesive cements. Conventional cements maintained near-neutral pH during initial curing, while self-adhesive cements exhibited lower initial pH (≈3.5) but neutralized to comparable levels (7.0–7.5) after light curing.

**Discussion:**

Resin cement bond strength developed significantly over time for all materials, with extended curing improving final bond strengths and adequate light curing accelerating pH neutralization in self-adhesive cements. Early bond strength was governed by individual material characteristics rather than cement category, though conventional cements surpassed self-adhesive systems by 2 h. Clinicians should prioritize extended light curing for thick restorations and advise patients to avoid occlusal loading during the early setting period, particularly with VA and PA.

## 1 Introduction

Lithium disilicate has become one of the most widely used CAD/CAM ceramic materials for indirect restorations, owing to its excellent esthetics, favorable mechanical properties, and predictable bonding behavior (1). As a silica-based ceramic, adhesion to lithium disilicate requires a two-step surface preparation: hydrofluoric acid etching to create micromechanical retention by selectively dissolving the glass matrix, followed by silanization to establish chemical coupling between the exposed silica phase and the organic matrix of the resin cement through siloxane bonds (2). Because lithium disilicate bonding is primarily silane-dependent, the integrity of the silane layer and the properties of the overlying resin cement are both critical to long-term restoration success (1). The choice of resin cement — and the ability to achieve adequate polymerization at the cement-ceramic interface — therefore plays a central role in determining clinical outcomes of lithium disilicate restorations, including retention, marginal seal, and overall durability (3–5).

Traditionally, dental professionals have relied on luting, a technique that uses cement-like materials for mechanical retention and sealing. However, luting has limitations in terms of bond strength and adaptability to different restoration materials (6, 7). The emergence of resin cements for bonding marked a significant advancement, offering improved adhesion, enhanced mechanical properties, and superior esthetics (8–10).

Resin cements are classified into two main categories based on their application technique and adhesive properties: conventional and self-adhesive resin cements. Conventional resin cements require a separate etching and bonding step, which allows for optimal adhesion to the tooth structure and various restorative materials (11–13). This multistep approach, while technique sensitive, consistently demonstrates superior bond strength and durability, particularly to dentin surfaces (13–15). The controlled conditioning process allows for optimal penetration of adhesive monomers into the pretreated tooth structure, resulting in a robust hybrid layer formation that provides excellent long-term adhesion.

In contrast, self-adhesive resin cements offer a simplified approach by incorporating acidic functional monomers that simultaneously demineralize and infiltrate the tooth structure, eliminating the need for separate conditioning steps (16). This streamlined procedure reduces technique sensitivity and chair time, making them attractive for routine clinical use. However, the simplified chemistry comes with trade-offs: self-adhesive cements generally exhibit lower initial bond strengths compared to conventional systems and demonstrate greater susceptibility to hydrolytic degradation over time (12, 15, 17, 18). Moreover, the demineralization process is less controlled compared to traditional etch-and-rinse or self-etch systems, often resulting in a shallower and more superficial interaction with dentin and enamel, which can lead to incomplete monomer penetration and weaker interfacial bonding, especially under challenging clinical conditions such as moisture or limited light access (19–21).

An additional consideration with dual-cure resin cements is the potential for chemical incompatibility between acidic adhesive components and the amine-based chemical initiator system embedded in these materials. The chemical polymerization of dual-cure cements relies on a redox initiation system comprising benzoyl peroxide and tertiary amines; however, the acidic monomers present in self-adhesive cements and self-etch adhesives can protonate and deactivate these amine initiators, thereby inhibiting the chemical curing component and reducing the overall degree of conversion at the cement–tooth interface (22). This interaction is particularly consequential in areas of limited light access — such as deep preparations, subgingival margins, or beneath opaque restorations — where chemical polymerization must compensate for insufficient light penetration. Incomplete polymerization resulting from this incompatibility may compromise interfacial bond strength, prolong the period of acidic monomer exposure to the pulp, and increase susceptibility to hydrolytic degradation over time. Understanding this mechanism is therefore clinically relevant when selecting cement systems and bonding protocols for lithium disilicate restorations.

Both cement types demonstrate excellent clinical performance compared to traditional luting cements. However, comprehensive evaluation of long-term clinical outcomes must consider procedural factors and clinical protocols. While dentists typically cement indirect restorations using either conventional or self-adhesive resin cements following manufacturer-guided protocols and bite adjustment procedures, the dual-cure polymerization mechanism of these materials presents unique clinical considerations due to extended setting times. Most dual-cure resin cements achieve substantial polymerization within the first hour after light activation, reaching approximately 66%–77% degree of conversion (DC), with complete setting and optimal mechanical properties typically achieved after 24 h when DC values can reach 75%–82% depending on the specific product and curing conditions (23, 24). Despite this well-documented time-dependent maturation process, evidence-based, time-specific clinical guidelines for post-cementation protocols remain lacking for individual products, potentially impacting optimal clinical outcomes.

This extended polymerization timeline introduces several clinical complications that warrant careful consideration. Prolonged and incomplete resin setting at the internal interface between vital tooth structure and indirect restorations may present concerns such as pulpal irritation and postoperative sensitivity, particularly in cases involving deep preparations (25–27). Additionally, the critical timing of restoration loading becomes paramount for both cement systems, as premature functional forces applied before complete polymerization can disrupt the developing polymer network and compromise long-term clinical success. The degree of conversion and mechanical properties such as flexural strength and modulus continue to develop after light exposure, with the most significant improvements occurring within the first day for dual-cure resin cements (23). Research indicates that longer light-curing times help maintain hardness and strength over time, reducing the negative effects of aging and water storage, particularly in deeper or less accessible regions (28). These multifaceted concerns highlight the complexity of achieving predictable outcomes with any resin cement system and underscore the importance of understanding their setting characteristics under various clinical conditions.

Given these considerations, although the importance of understanding initial bonding strength and setting characteristics of resin cements is well recognized, there remains a significant gap in comprehensive studies investigating these factors at various time points, including immediately after curing and at final setting times. Moreover, the potential effects of pH changes during the setting process on pulp vitality have not been thoroughly examined.

Therefore, this study aimed to address these knowledge gaps by examining the shear bond strengths of conventional and self-adhesive resin cements bonded to lithium disilicate at different curing and setting times, simulating both immediate and delayed use of restorations. Additionally, we investigated the pH changes during the recommended setting time to assess the potential effects on pulp vitality. Thus, we hypothesized that (1) extended light-curing time would improve bond strength, (2) adequate light curing would accelerate pH neutralization in self-adhesive cements compared to no light curing, and (3) conventional cements would achieve higher initial bond strengths compared to self-adhesive systems.

## 2 Materials and methods

**Figure 1:**
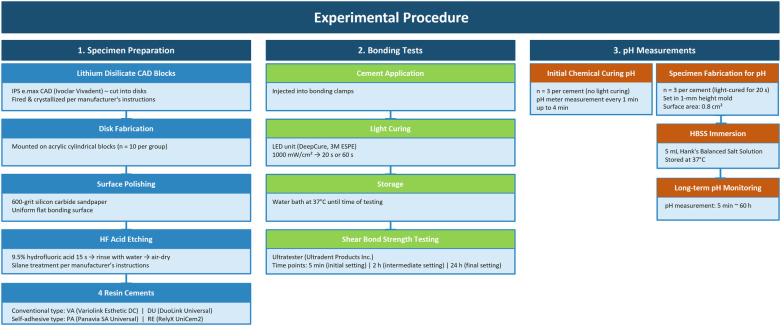
Schematic diagram of the experimental procedure illustrating specimen preparation, bonding tests, and pH measurements.

Lithium disilicate CAD blocks (IPS e.max CAD, Ivoclar Vivadent, Schann, Liechtenstein) were cut into disks, fired and crystallized according to the manufacturer's instructions (*n* = 10/group). Disks were mounted on acrylic cylindrical blocks (Great Lakes Dental Technologies, Tonawanda, NY, USA) The disk surfaces were polished using a 600-grit silicon carbide sandpaper. The polished surfaces were etched with 9.5% hydrofluoric acid (BISCO Inc., Schaumburg, IL, USA) for 15 s, thoroughly rinsed with water, and air-dried. Silane coating (Porcelain Primer, BISCO Inc.) was applied following the manufacturer's instructions. Four resin cements in Table 1 were tested: Variolink Esthetic DC (VA; Ivoclar Vivadent), DuoLink Universal (DU; BISCO Inc.), Panavia SA Universal (PA; Kuraray Noritake Dental Inc.), and RelyX UniCem2 (RE; 3M ESPE). The overall experimental procedure is illustrated in Figure 1.

**Table 1:** Resin cements used in this study.

Product name	Abbreviation	Company	Classification	Main Components
Variolink Esthetic DC	VA	Ivoclar Vivadent, Schaan, Liechtenstein	Conventional resin cement	UDMA, proprietary methacrylates, Ivocerin, thiocarbamide, hydrogen peroxide, copper ions, spherical barium glass, ytterbium trifluoride, silica, pigments, stabilizers, rheology modifiers
DuoLink Universal	DU	BISCO Inc., Schaumberg, IL, USA	Conventional resin cement	Bis-GMA, UDMA, TEGDMA, BPO, CQ, amines, barium glass, silica, ytterbium fluoride, pigments, stabilizers, rheology modifiers
Panavia SA Universal	PA	Kuraray Noritake Dental Inc.	Self-adhesive resin cement	MDP, LCSi, proprietary methacrylates, BPO, CQ, amines, inorganic fillers (62 wt%), sodium fluoride, pigments, stabilizers, rheology modifiers
RelyX UniCem2	RE	3M ESPE	Self-adhesive resin cement	Phosphoric acid methacrylate, proprietary methacrylates, BPO, CQ, amines, silanated silica, barium glass, other fillers (72 wt%), pigments, stabilizers, rheology modifiers

Bis-GMA, bisphenol A-glycidyl methacrylate; UDMA, urethane dimethacrylate; TEGDMA, triethylene glycol dimethacrylate; MDP, 10-methacryloyloxydecyl dihydrogen phosphate; BPO, benzoyl peroxide; CQ, camphorquinone, LCSi, long-chain silane.

For bonding tests, cements were injected into bonding clamps (Ultradent Products Inc., South Jordan, UT, USA) and light-cured using an LED unit (DeepCure; 3M ESPE, St. Paul, MN, USA) at 1,000 mW/cm^2^ for either 20 or 60 s. The samples were stored in a water bath at 37 °C until testing, and shear bond strength was measured at 5 min, 2 h, and 24 h post-cementation using an Ultratester (Ultradent Products Inc.).

The pH level of each cement (*n* = 3 per cement) without light curing was measured during the initial 4 min after dispensing using a calibrated pH meter (AquaSearcher AB33PH; Ohaus Corp., Parsippany, NJ, USA). Additionally, cement samples (surface area of 0.8 cm^2^) were fabricated in a 1-mm height mold by light-curing for 20 s, placed in 5 mL HBSS (Hank's Balanced Salt Solution; Sigma-Aldrich, St. Louis, MO, USA), and pH was measured at intervals from 5 min to 60 h while samples were stored at 37 °C.

Data for 20- and 60-second curing at different storage times were analyzed using a mixed-effects model (REML) with Sidak's multiple comparisons test in the latest version of GraphPad Prism (GraphPad Software, Boston, MA, USA). This approach was chosen to account for missing values resulting from initial bond failures and to appropriately address the unbalanced design caused by 14 missing data points. Statistical significance was set at *P* = 0.05.

## 3 Results

**Figure 2:**
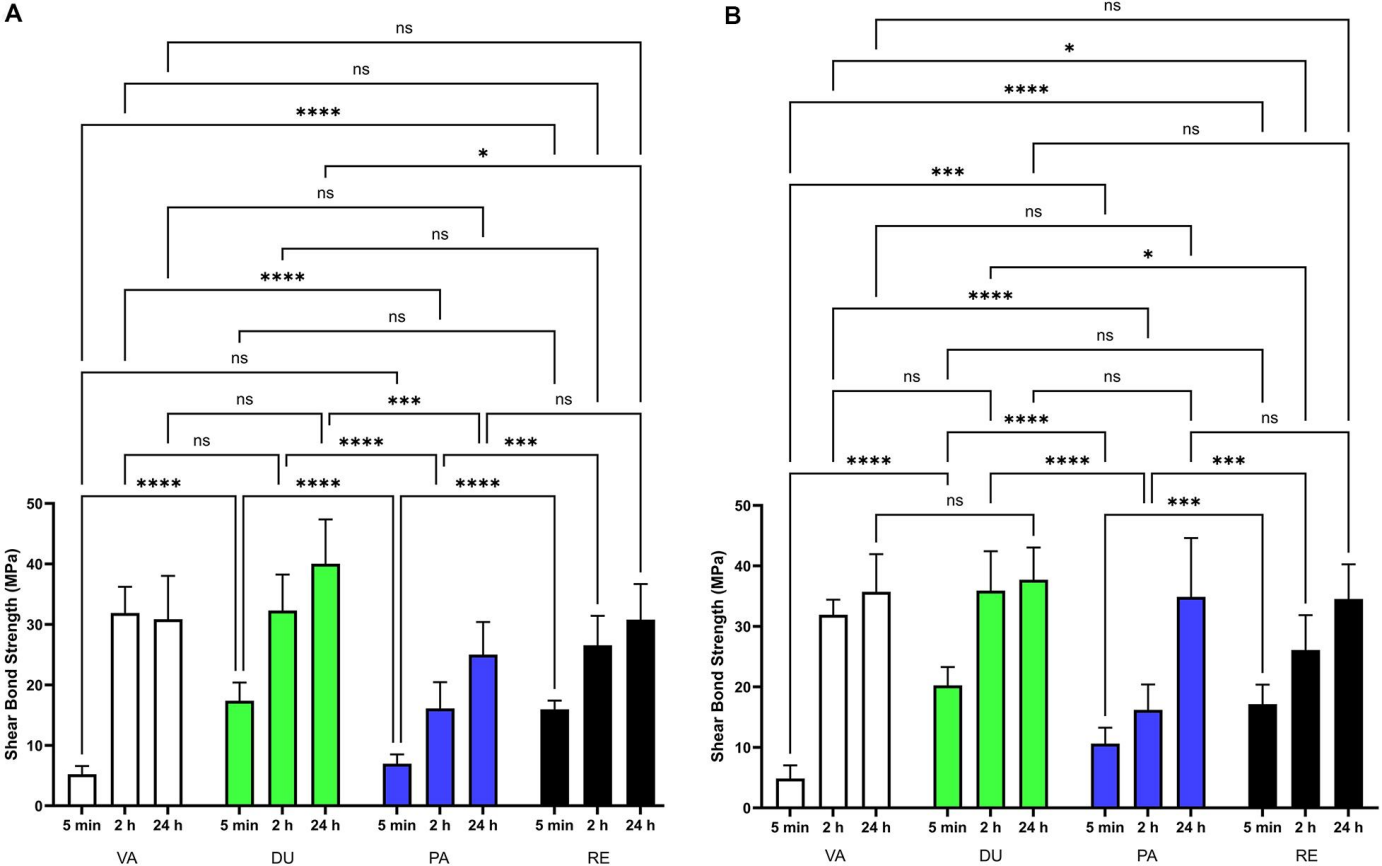
Time-dependent shear bond strength development of various resin cements with different light-curing durations. **(A)** 20-second light-curing time and **(B)** 60-second light-curing time. Measurements were taken at 5 min, 2 h, and 24 h after initial setting for each cement type.

The shear bond strengths of four dental resin cements (VA, DU, PA, and RE) were measured at 5 min, 2 h, and 24 h intervals under both 20 s and 60 s curing conditions (Figure 2, Tables 2A,B, 3). The mixed-effects analysis revealed significant effects for all three fixed factors as shown in Table 2A: time relapse, material, and their interaction for both 20 s curing [F(1.491, 83.51) = 184.8, F(3, 112) = 46.43, and F(6, 112) = 7.230, respectively; all *P* < 0.0001] and 60 s curing [F(1.491, 87.17) = 238.0, F(3, 118) = 48.63, and F(6, 118) = 12.40, respectively; all *P* < 0.0001].

**Table 2:** Mixed-Effects analysis results and material performance.

A. Mixed-Effects Analysis Results		
Fixed Effects	20-second curing	60-second curing
Time relapse	F(1.491, 83.51) = 184.8****	F(1.491, 87.17) = 238.0****
Material	F(3, 112) = 46.43****	F(3, 118) = 48.63****
Time × Material	F(6, 112) = 7.230****	F(6, 118) = 12.40****

B. Material Performance (Mean ± SD in MPa)				
Material	Curing time	Bond Strength (MPa)		
		5 min	2 h	24 h
VA	20 s	7.86 ± 5.69	31.90 ± 4.34	32.69 ± 7.73
	60 s	5.72 ± 3.51	34.46 ± 5.54	36.59 ± 11.01
DU	20 s	17.17 ± 3.28	32.90 ± 6.04	34.73 ± 10.92
	60 s	24.18 ± 11.08	37.29 ± 3.68	37.00 ± 11.83
PA	20 s	6.98 ± 1.53	20.26 ± 6.26	27.30 ± 6.88
	60 s	15.22 ± 6.34	29.79 ± 5.99	32.37 ± 8.02
RE	20 s	15.96 ± 1.44	22.75 ± 7.76	29.85 ± 5.32
	60 s	6.91 ± 1.06	36.88 ± 4.81	35.96 ± 5.70

All fixed effects significant at **** *P* < 0.0001.

**Table 3:** Summary of significant pairwise comparisons.

Comparison type	20-second curing	60-second curing
**Material comparisons at 5 min**		
Significant differences	VA < DU****; VA < RE****	VA < DU****; VA < PA****; DU > PA***; DU > RE****; PA > RE****
**Material comparisons at 2 h**		
Significant differences	VA > PA****; VA > RE****; DU > PA****; DU > RE**; PA < RE***	VA > PA****; DU > PA****; PA < RE****
**Material comparisons at 24 h**		
Significant differences	None	VA < RE***; PA < RE**
**Time point comparisons**		
All materials	5 min < 2 h****; 5 min < 24 h****	5 min < 2 h****; 5 min < 24 h****
Unique differences	RE: 2 h < 24 h***	None between 2 h and 24 h
**Overall rankings**		
Best to worst	DU > RE > VA > PA	DU > RE > VA > PA
Greatest improvement with 60-second curing	—	VA (+3%); DU (+15%); PA (+6%); RE (+10%)

Statistical Notes: All comparisons performed using Sidak's multiple comparisons test.

Significance levels: **** *P* < 0.0001; *** *P* < 0.001; ** *P* < 0.01; * *P* < 0.05.

Analysis based on REML mixed-effects model.

Sample: 124 observations, 46 subjects, 14 missing values.

Table 2B presents the mean and standard deviation values for each material at each time point. Under 20 s curing conditions (Figure 2A), pairwise comparisons detailed in Table 3 showed that at 5 min, VA had significantly lower bond strength than DU and RE (*P* < 0.0001). At 2 h, VA and DU showed significantly higher strength than PA and RE (*P* < 0.0001), while DU showed greater strength than RE (*P* < 0.01), and PA showed lower strength than RE (*P* < 0.001). At 24 h, no significant differences were observed between materials.

For 60 s curing (Figure 2B), Table 3 shows that at 5 min, VA showed significantly lower strength than DU and PA (*P* < 0.0001), while DU was significantly stronger than PA (*P* < 0.001) and RE (*P* < 0.0001), and PA was significantly stronger than RE (*P* < 0.0001). At 2 h, VA and DU were significantly stronger than PA (*P* < 0.0001), and PA was significantly weaker than RE (*P* < 0.0001). At 24 h, VA and PA showed significantly lower strength than RE (*P* < 0.001 and *P* < 0.01, respectively).

For time comparisons, Table 3 indicates that all materials showed significant increases from 5 min to 2 h and from 5 min to 24 h (*P* < 0.0001) in both curing protocols. With 20 s curing, only RE showed a significant increase from 2 h to 24 h (*P* < 0.001), while with 60 s curing, no materials showed significant changes between 2 h and 24 h.

**Figure 3:**
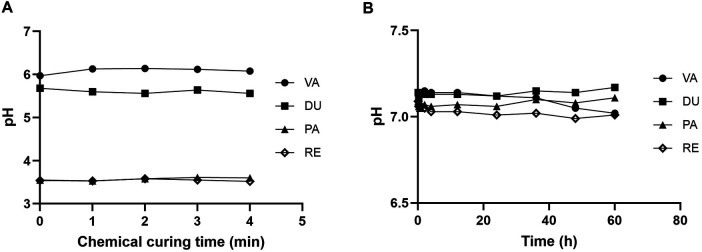
pH dynamics of resin cements during the setting process. **(A)** Initial pH changes of freshly mixed resin cements during the first 4 min of chemical curing. **(B)** Long-term pH changes of set cements immersed in HBSS (Hank's Balanced Salt Solution) over 60 h.

The pH changes (average values) of the four resin cements were monitored during initial chemical curing and after setting for 60 h (Figure 3). During initial chemical curing (Figure 3A), VA maintained the highest pH values (consistently above 6.0), while DU showed slightly lower values (between 5.5 and 6.0). PA and RE exhibited considerably lower pH levels (approximately 3.5). All materials maintained relatively stable pH values with minimal fluctuations during the 4 min period. Following initial chemical and light curing processes, all materials demonstrated elevated pH values compared to their initial state, ranging between 7.0 and 7.5 (Figure 3B). VA and DU maintained slightly higher pH values than PA and RE. While all materials showed a minor pH decrease over the 24-hour period, their overall pH levels remained relatively stable.

## 4 Discussion

This study offers valuable insights into the behavior of conventional and self-adhesive resin cements during the critical early and late phases of the setting process. While clinicians take precautions during the cementation of indirect restorations, unavoidable situations may arise in which patients inadvertently apply dynamic forces or experience temperature changes. These factors can compromise the long-term stability of restorations, leading to sensitivity or other signs of clinical failure.

All tested cements exhibited a significant increase in bond strength over the 24 h period, irrespective of the curing time. This finding is consistent with those of previous studies and highlights the crucial role of the post-curing period in achieving the optimal bond strength. The ongoing increase in bond strength can be attributed to factors such as continuous polymerization, stress relaxation, and minimal water sorption by the resin matrix (29–32).

A trend was observed in which all resin cements showed slight improvements in the final bond strength with extended curing time (60 s) compared to shorter curing time (20 s), although the differences were not statistically significant. This can be explained by the fact that the depth of the bonding mold is approximately 4 mm, and a 20 s irradiation time may not be sufficient for complete light curing (33, 34). It is proved that dual-cure resin cements can achieve significantly higher shear bond strength with adequate light-curing time, not just with chemical curing alone, and sufficient light irradiation results in faster setting times (35, 36, 1). Moreover, conventional resin cements (VA and DU) achieved their maximum bond strengths within 2 h of the setting process, under a sufficient curing time (60 s), faster than self-adhesive resin cements (PA and RE) in Figure 2B. Considering that the use of indirect restorations cannot be guaranteed for several hours the setting process, conventional resin cement might provide more stable clinical results within a shorter timeframe.

The variation in the early bond strength development among cements has important clinical implications. Cements with higher initial bond strengths may be preferable in situations requiring immediate stability, whereas the significant increase in bond strength from 5 min to 2 h for all cements suggests that delaying occlusal loading, when possible, could be beneficial. During the early chemical and light curing processes (< 5 min), DU and RE had significantly higher bond strengths, regardless of the curing time. Thus, VA and PA require special caution in the clinic after the bonding process is complete. Clinicians should be aware of the potential for lower bond strength during the early stages and take appropriate precautions to ensure restoration stability.

The pH dynamics observed in this study highlight a notable difference between conventional (VA and DU) and self-adhesive (PA and RE) cements. Conventional cements maintained a near-neutral pH during the initial 4 min period, while self-adhesive cements initially exhibited significantly lower pH levels. This variation can be attributed to the chemistry of self-adhesive cements, which contain acidic monomers that facilitate self-etching. Although the low initial pH of self-adhesive cements may raise concerns regarding potential pulpal irritation, the pH is designed to neutralize over time (37, 38). As shown in Figure 3B, self-adhesive resin cements, when light-cured, achieve neutral pH levels comparable to those of conventional resin cements.

Moreover, self-adhesive cements, which incompletely remove the smear layer, neutralize their initial acidity during the setting process, potentially reducing postoperative tooth sensitivity compared to etch-and-rinse systems (39, 40). However, adequate light curing is essential for proper activation of acidic monomers and timely pH neutralization. Given the restoration thickness and consequent reduced light penetration, high-intensity light curing becomes more critical for self-adhesive systems. Insufficient curing may result in delayed pH neutralization and compromised bond strength, particularly since shorter light-curing times produce lower initial strength that is not fully compensated by subsequent 24-hour post-cure polymerization (41).

The time-dependent bond strength development and pH dynamics observed across all four cements can be further understood through three interrelated physicochemical parameters: degree of conversion (DC), water sorption, and depth of polymerization. DC — the proportion of methacrylate double bonds converted to polymer chains during curing — is the primary driver of mechanical property development in resin cements. Higher DC values are associated with improved flexural strength, reduced residual monomer release, and greater chemical stability (23, 24). Dual-cure resin cements typically achieve DC values of approximately 66%–82% within 24 h (23, 24), with the most significant gains occurring in the first two hours after light activation — a pattern that directly explains the steep bond strength increase from 5 min to 2 h observed for all materials in this study. Notably, the self-adhesive cements PA and RE showed lower initial bond strengths, which aligns with their acidic monomer chemistry: until sufficient DC is achieved, unreacted acidic monomers persist at the interface, simultaneously depressing pH and limiting mechanical strength development. This was reflected in the lower initial pH values (≈3.5) recorded for PA and RE during the first 4 min of setting. Depth of polymerization is particularly relevant in the clinical context of cemented indirect restorations, where restoration thickness attenuates light transmission and restricts the zone of adequate curing at the cement-tooth interface. In our experimental model, the bonding mold depth of approximately 4 mm represents a challenging light-penetration scenario, and the improved bond strengths observed with 60 s curing — most pronounced for DU (+15%) and RE (+10%) — suggest that extended irradiation meaningfully increased DC at depth. Water sorption adds a longer-term dimension to these findings: partially polymerized resin matrices with lower DC absorb more water, which accelerates hydrolytic degradation of both the polymer network and the ceramic-resin interface over time (18, 21). Extended light curing, by increasing DC and reducing residual monomer content, therefore not only improves immediate bond strength but also confers greater resistance to water-driven degradation — an important consideration for the long-term durability of lithium disilicate restorations.

Although informative, this study has several limitations that should be acknowledged. First, we investigated a limited number of resin cements, which may not fully represent the diverse range of products available on the market. Second, the time intervals chosen for measurement, while providing valuable data, did not include various intermediate points, such as 30 min or 1 h, which could have offered a more comprehensive view of the cement's behavior during the critical early setting phases. Third, the study lacked long-term data under simulated oral conditions, such as fatigue testing with indirect restorations cemented to teeth, which would more closely mimic real-world clinical scenarios. Furthermore, efforts are needed to connect these *in vitro* findings to clinical data to enhance their relevance and applicability in dental practice. Finally, testing a broader range of mechanical, physical, and chemical properties can provide a more comprehensive understanding of the early and long-term stability of these cements during the cementation process.

Within the limitations of the current study, hypotheses (1) and (2) were fully supported, and hypothesis (3) was partially supported. Extended light-curing time (60 s vs. 20 s) improved final bond strengths across all materials, and adequate light curing (20 s) accelerated pH neutralization in self-adhesive cements compared to no light curing. Regarding hypothesis (3), early bond strength at 5 min was determined more by individual material characteristics than by cement category: DU (conventional) and RE (self-adhesive) both demonstrated significantly higher bond strengths than VA (conventional) and PA (self-adhesive), indicating that the initial strength advantage was material-specific rather than a consistent class effect. However, by 2 h, conventional cements (VA and DU) outperformed self-adhesive cements, providing partial support for the hypothesis. These findings have direct clinical implications. Clinicians should consider using extended light-curing times whenever restoration geometry or thickness limits light penetration, as this not only maximizes immediate bond strength but also promotes more complete DC — reducing long-term susceptibility to water sorption and interfacial degradation. Given the significantly lower bond strengths observed at 5 min for VA and PA in particular, patients should be counseled to avoid occlusal loading immediately after cementation, particularly with these materials. For self-adhesive cements, adequate light curing is especially critical: without it, residual acidic monomers remain at the interface, delaying pH neutralization and compromising early bond development. Taken together, these results support cement-specific post-cementation protocols rather than a one-size-fits-all approach, and underscore the importance of educating patients about the significance of a protected non-loading period following indirect restoration placement.

## 5 Conclusion

All resin cements showed significant time-dependent bond strength development, with extended light curing enhancing final bond strengths and adequate polymerization promoting timely pH neutralization in self-adhesive systems. Initial bond strength at 5 min reflected individual material behavior rather than cement type, whereas conventional cements outperformed self-adhesive systems by 2 h. Clinically, extended light curing should be prioritized especially for thick restorations, and patients should be advised to refrain from occlusal loading immediately after cementation, particularly when VA and PA are used.

## Data Availability

The raw data supporting the conclusions of this article will be made available by the authors, without undue reservation.
